# Modification of carbon felt anodes using double-oxidant HNO_3_/H_2_O_2_ for application in microbial fuel cells

**DOI:** 10.1039/c7ra12923h

**Published:** 2018-01-09

**Authors:** Yu Zhao, Yan Ma, Ting Li, Zhishuai Dong, Yuxue Wang

**Affiliations:** College of Chemistry and Chemical Engineering, Taiyuan University of Technology Taiyuan 030024 PR China zhaoyu@tyut.edu.cn tyut1995@126.com

## Abstract

Carbon felt is widely used as an anode material in microbial fuel cells (MFCs) because of its high specific surface area, low cost, good electrical conductivity, and biocompatibility. In this paper, carbon felt samples were thermally treated with a mixed solution of concentrated HNO_3_ and 30% H_2_O_2_ with different volume ratios of 1 : 3 (MFC-1), 1 : 1 (MFC-2), and 3 : 1 (MFC-3). The electrochemical performance of the resulting MFCs were investigated by cyclic voltammetry, electrochemical impedance spectroscopy, chronoamperometry and polarization curve measurement. Fourier transform infrared spectroscopy and scanning electron microscopy were conducted to characterize the functional groups and the morphology of the carbon felts. After modification, the number of oxygen-containing functional groups in MFC-1, MFC-2, and MFC-3 increased compared with MFC-4 (bare anode MFC), the start-up time of the obtained MFCs was markedly shortened, and the charge transfer resistance of the bioanode was decreased. In MFC-2, the maximum power density was 758.2 mW m^−2^, which was 51.1% higher than MFC-4. Increases of oxygen-containing functional groups on the modified anodes favored the adsorption and growth of bacteria and acceleration of electron transport between the electrode and bacteria. Thus, the electrochemical characteristics of MFCs employing these anodes were improved.

## Abstract 2

Carbon felt is widely used as an anode material in microbial fuel cells (MFCs) because of its high specific surface area, low cost, good electrical conductivity, and biocompatibility.

## Graphical abstract

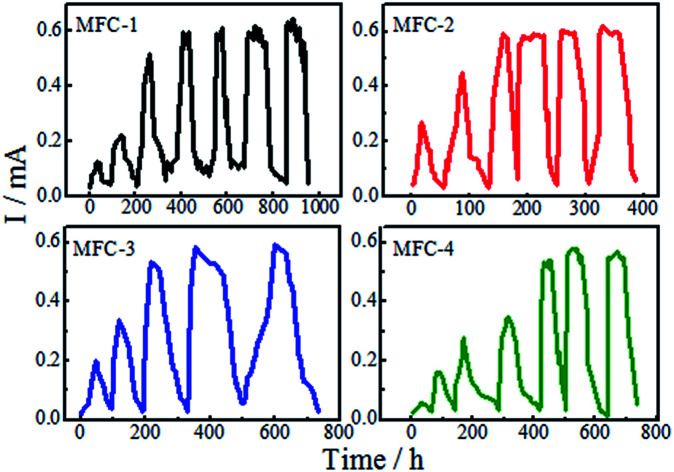

## Introduction

Microbial fuel cells (MFCs) are considered as a promising technology in the field of renewable energy production and wastewater treatment. In these cells, exoelectrogenic bacteria directly converted chemical energy stored in organic waste into electrical energy.^1–3^ Despite their obvious benefits, however, MFCs suffered from low energy production and poor organic degradation. Thus, the performance of MFCs should be improved for practical application, which was influenced by several factors, including microbial species, types and properties of the substrate, diaphragm material, electrode material, and system design.^4–7^

The electrode material, especially anode material, played a crucial role in the power production of MFCs. The electrode material provided support for exoelectrogenic bacterial attachment and promoted the export of electrons generated in the redox reaction. Carbon materials, such as carbon paper, carbon cloth, carbon felt, carbon fiber brush, graphite rods, and graphite plates, were the most widely used anode materials in MFC. High-performance anode materials should be inexpensive and presented good hydrophilicity, large specific surface area, good conductivity, excellent electrochemical properties, and good biocompatibility.^8–11^ Several studies had shown that surface modification of anode materials mainly affected the electron transfer mechanism in two ways. First, changes in the structure of the material providing additional areas for bacterial adhesion. Second, the presence of functional groups favored electron transfer between the bacteria and electrodes.^12^

According to Mohamed *et al.*,^13^ the power generation of MFC was significantly affected by doping superficial nitrogen groups on the anode surfaces of carbon cloth and carbon paper. From Huang *et al.*,^14^ after redox mediator modifying anodes prepared by electrodepositing riboflavin and humic acid on the surface of graphite felt, MFC exhibited excellent electrocatalysis activity and showed decrease in internal resistance along with increase in maximum power density. Kang *et al.*^15^ observed that electrochemical characteristics of MFC was enhanced through utilizing conductive polymer onto graphite felt base anodes. Li *et al.*^16^ demonstrated that the performance of MFC was improved after modifying carbon felt anode using two conductive polymer materials, polyaniline and poly(aniline-*co-o*-aminophenol).

This paper presented a new strategy to chemically treat carbon felts using mixed solutions of concentrated HNO_3_ and 30% H_2_O_2_ at different volume ratios. The morphology, hydrophilicity, and electrochemical properties of the treated carbon felts were characterized.

## Materials and method

### Preparation of electrodes

Carbon felts (Shanghai Lishuo Composite Material Technology Co. Ltd., Shanghai, China) with a 2 × 5 cm^2^ geometric area were treated with different proportions of mixed HNO_3_ and H_2_O_2_. The concentration of concentrated HNO_3_ and H_2_O_2_ was 16 mol L^−1^ and 8.8 mol L^−1^, respectively. The carbon felts were submerged in 200 mL of mixed solution of double oxidant, ultrasonically dispersed at room temperature for 0.5 h, then heated in air at 450 °C for 0.5 h in a muffle furnace. The resultant samples were washed repeatedly with deionized water until a constant pH was achieved. The samples were dried at 60 °C overnight to obtain the modified anode material. MFC-4 referred to the bare anode MFC, in which carbon felt anode was not modified using double-oxidant HNO_3_/H_2_O_2_. Table 1 showed the treatment conditions of each sample.

**Table 1:** Treatment conditions of carbon felts

Sample	*V*_HNO_3__ (mL)	*V*_H_2_O_2__ (mL)	*V*_HNO_3__ : *V*_H_2_O_2__
MFC-1	50	150	1 : 3
MFC-2	100	100	1 : 1
MFC-3	150	50	3 : 1

### MFC construction and operation

The cathode and anode chambers of the MFCs were of similar geometries, and their effective volume was also 80 mL. The two chambers were separated by a proton exchange membrane (Nafion 115, DuPont) with an area of 4 cm^2^. The carbon felt (2 × 4 cm^2^) served as anode, and a stainless steel mesh (2 × 2 cm^2^, 99.99%) served as cathode. The external circuit was connected to a constant external resistance of 1000 Ω, and the anode chamber was sealed to cut off air. Thus, an anaerobic environment was maintained in MFC. The sludge came from a local domestic sewage treatment plant in Taiyuan, China.

The anolyte composition: glucose (0.011 mol L^−1^), potassium chloride (0.0017 mol L^−1^), ammonium sulphate (0.0042 mol L^−1^), sulfuric acid (0.0008 mol L^−1^), calcium chloride (0.00014 mol L^−1^), ferric chloride (0.0000037 mol L^−1^), manganese sulfate (0.000118 mol L^−1^), sodium bicarbonate (0.03726 mol L^−1^), disodium hydrogen phosphate dodecahydrate (0.0383 mol L^−1^), sodium dihydrogen phosphate dihydrate (0.0617 mol L^−1^). The catholyte composition: potassium ferricyanide (0.1 mol L^−1^), disodium hydrogen phosphate dodecahydrate (0.0383 mol L^−1^), sodium dihydrogen phosphate dihydrate (0.0617 mol L^−1^). Glucose served as substrate in anode chamber, potassium ferricyanide acted as electron acceptor in cathodic chamber.

The MFCs were operated in batch mode. When the output current was less than 0.02 mA, the substrate was replaced. When the output maximum current reached a stable value, a mature biofilm was formed and the battery was started up stably.

### Analyses and calculations

All electrochemical tests were performed using a multichannel potentiostat (Princeton VMP III, US) with a three-electrode system consisting of a working electrode, a saturated calomel electrode (SCE) as reference electrode, and a stainless steel mesh electrode as cathode. Cyclic voltammetry (CV) was performed by applying a potential ramp at a scan rate of 5 mV s^−1^ over the potential range from −0.5 V to 0.5 V to the working electrode. The electrochemical impedance of anode was measured at frequencies ranging from 100.000 kHz to 5.000 mHz with a potential amplitude of 10 mV. The EIS tests were conducted at open circuit condition. Scanning data were fitted and simulated using ZSimpWin 3.10 software (Echem). Chronoamperometry was performed at a constant potential of −0.3 V (*vs.* SCE). Polarization curve measurements were obtained at 20 mV s^−1^ within a certain potential range. The performance of the fuel cells was critically evaluated based on power output. The power density curves were obtained by varying the external resistances. Current production during steadily operating of fuel cell was monitored by connecting to various external resistances (100 Ω to 100 kΩ) using a multimeter. Power output (mW) was calculated using the equation *P* = *IU*. Power density (mW m^−2^) and current density (mA m^−2^) were calculated as a function of the anodic surface area (m^2^). Fourier transform infrared spectroscopy (FTIR) was performed to analyze the functional groups formed on the electrochemically oxidized carbon felts. Treated felts (2 mg) were cut and mixed with KBr (200 mg), and the samples obtained were analyzed by a FTIR spectrometer (Nicolet is 5, US). Scanning electron microscopy (JSM-7001F, JEOL, Japan) was performed to analyze the bacterial morphology. Water contact angle measurement (Phoenix-300, Korea) was used to analyze the hydrophilic nature and the hydrophobic nature.

## Results and discussion

### Acclimation of MFC

**Fig. 1:**
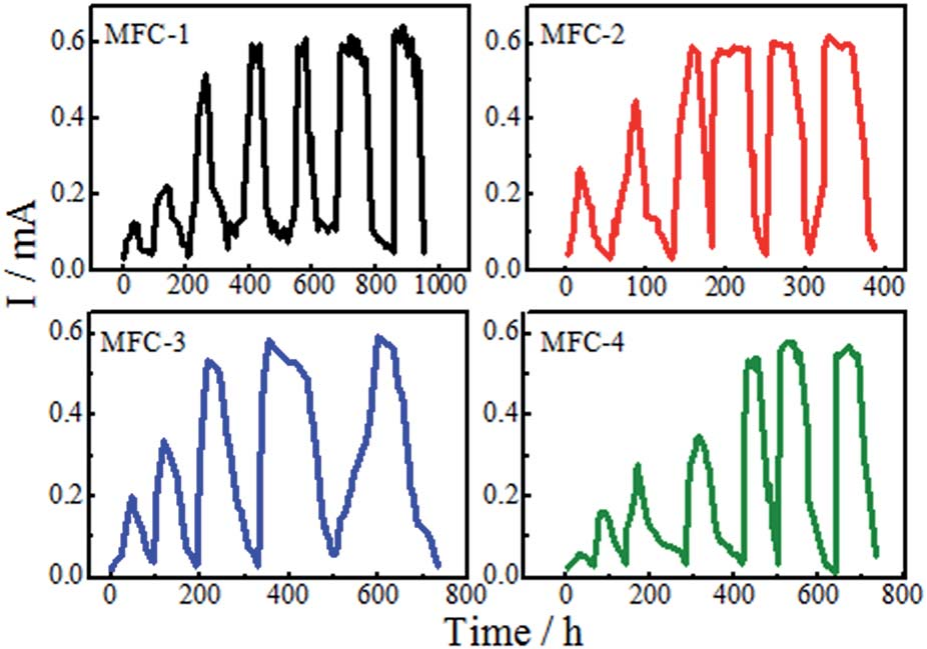
Start-up of MFCs.

After inoculation, the MFCs took batch operation mode, each cell had different operating period. As shown from Fig. 1, the time to form the mature biofilm were as follows: MFC-1, 400 h; MFC-2, 160 h; MFC-3, 220 h; and MFC-4, 450 h. The time required by the MFCs to reach the peak power output was shortened by anode modification. Modifying with a mixed solution of HNO_3_ and H_2_O_2_ could evidently help reduce the start-up time of the MFCs, thereby improving their electrochemical performance. Compared with mixed solutions of other ratios, the mixed solution with a volume ratio of 1 : 1 (MFC-2) was the most effective in improving the biochemical properties of carbon felt. These results were due to enhancements in the specific surface area of anode caused by acid-induced surface modification.^17^ Acid treating increased the roughness of anode surface and provided a more conductive environment for microbial reproduction. The modifying hastened biofilm formation on carbon felt surfaces, effectively enhanced the power outputs.^18^ Increases of –OH and –COOH (Fig. 7) on the modified carbon felt benefited the adhesion and reproduction of bacteria, thereby enabling rapid formation of mature biofilms.

### Electrochemical measurement

#### Cyclic voltammetry

**Fig. 2:**
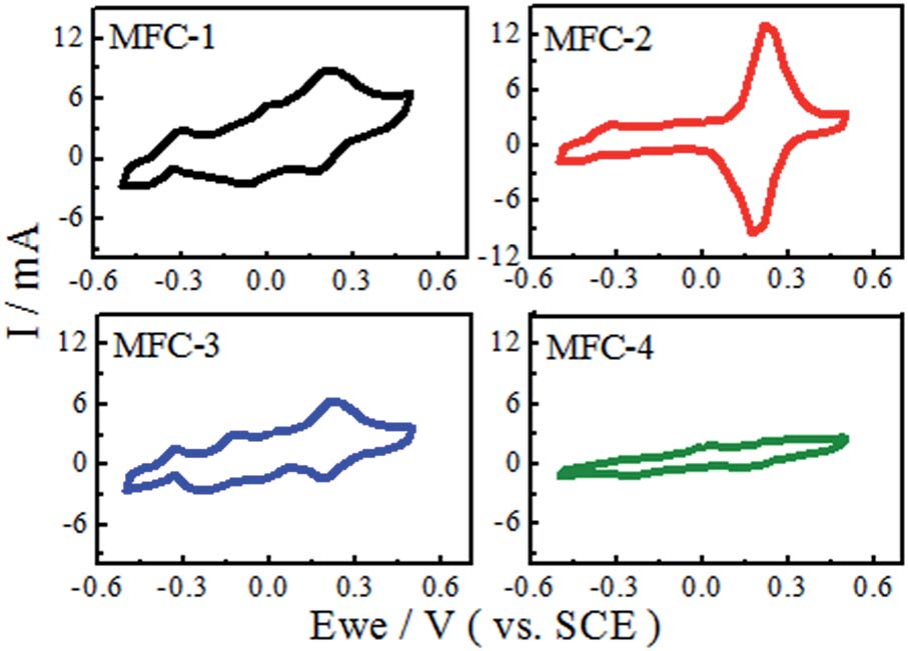
Cyclic voltammograms of MFCs.

The electrochemical behavior of the modified carbon felts was characterized by CV (Fig. 2). The cyclic voltammograms of MFC-1 and MFC-3 showed two pairs of reversible redox peaks, while MFC-2 presented a single pair of reversible redox peaks. The redox peaks of MFC-4 were especially weak. These results suggested that multistep electrochemical reaction took place on the biofilm. Different functional groups on the modified carbon felt anode surfaces directly affected the positions and sizes of the redox peaks. The weak redox peaks of MFC-4 indicated that the electrochemical activity of biofilm was low. Evident redox peaks were observed in MFC-1, MFC-2, and MFC-3. The two pairs of redox peaks of MFC-1 were observed at (0.21 mV, 8.85 mA; 0.17 mV, −1.67 mA) and at (0.005 mV, 5.47 mA; −0.058 mV, −2.54 mA); there was another oxidation peak (−0.31 mV, 2.81 mA). The two pairs of redox peaks of MFC-3 were observed at (0.21 mV, 6.33 mA; 0.18 mV, −1.35 mA) and at (−0.14 mV, 3.00 mA; −0.017 mV, −1.53 mA); there was also another oxidation peak (−0.33 mV, 1.69 mA). These characteristics indicated that the mechanism of electrochemical reaction occurring on the biofilm was extremely similar in MFC-1 and MFC-3, while strong and reversible electrochemical oxidation and reduction reaction took place on the biofilms. The bioelectrochemical reaction occurring was multi-step reaction. MFC-2 showed a pair of redox peaks at (0.21 mV, 13.00 mA; 0.17 mV, −9.54 mA), this might be attributed that two large redox peaks were superimposed into a single peak, in particular the peak current reached 13.00 mA. This meant that electrochemical activity of the bacteria on the modified felts was the highest among all MFCs. Differences between the observed redox peaks among the samples could be attributed to differences of the volume ratio of HNO_3_ and H_2_O_2_. The much higher electrochemical activity might have resulted from enhancements of electron transmit between the bacteria and the modified carbon felts, the increased attachment of bacteria on the treated electrodes might also explained the results.^19^

#### AC impedance

**Fig. 3:**
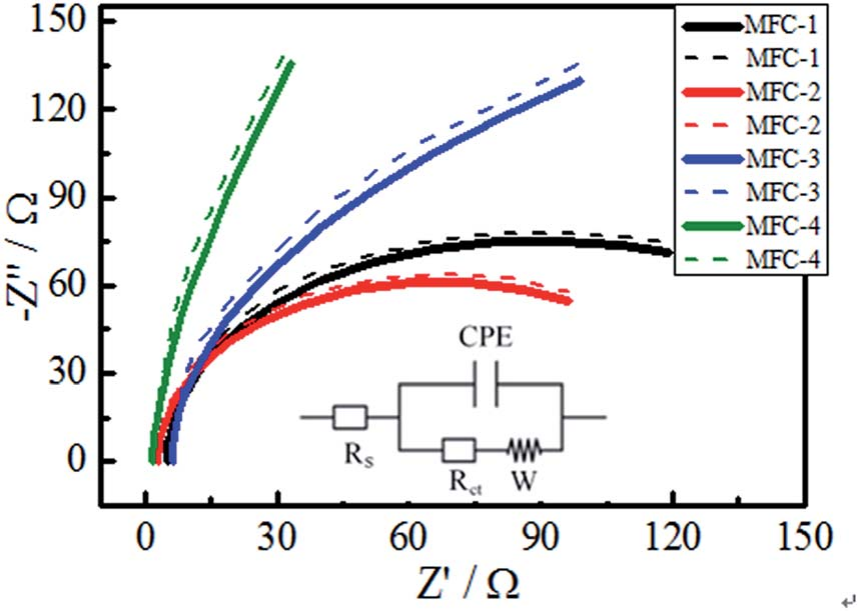
Nyquist plot of MFCs (dotted line, experimental curve; solid line, fitting curve).

The Nyquist plots were shown in Fig. 3. The experimental spectra was fit into an equivalent circuit according to Wagner to estimate the impedance data quantitatively.^20^ The inset figure in Fig. 3 showed an equivalent circuit consisted of a solution resistance, followed by Randles-type charge transfer resistance, a Warburg diffusion resistance, and a constant phase element (CPE). A CPE suggests a rough electrode surface, which is used to simulate the non-ideal behavior of a distributed capacitor. The experimental spectra were fitted and simulated by ZSimpWin 3.10 software (Echem). The charge-transfer resistance dominated the internal resistance of bioanodes. Table 2 showed that charge-transfer resistances of MFC-1, MFC-2, MFC-3, and MFC-4 were 130.11, 115.72, 116.26 and 148.23 Ω, respectively, likely because the double-oxidant modifying increased the C=C groups of the carbon felt, which could hasten the electron transfer rate on carbon felts. Meanwhile oxygen-containing functional groups on the treated carbon felts also increased such as biocompatibility, specific surface area, and the number of exoelectrogenic bacteria.

**Table 2:** Impedance fitting of the different MFCs

No.	*R*_s_ (Ω)	*Q* (Ω)	*R*_ct_ (Ω)	*W* (Ω)
MFC-1	4.90	0.09	130.11	0.16
MFC-2	1.79	0.09	115.72	0.01
MFC-3	6.18	0.03	116.26	0.02
MFC-4	3.06	0.13	148.23	0.49

#### Chronoamperometry

**Fig. 4:**
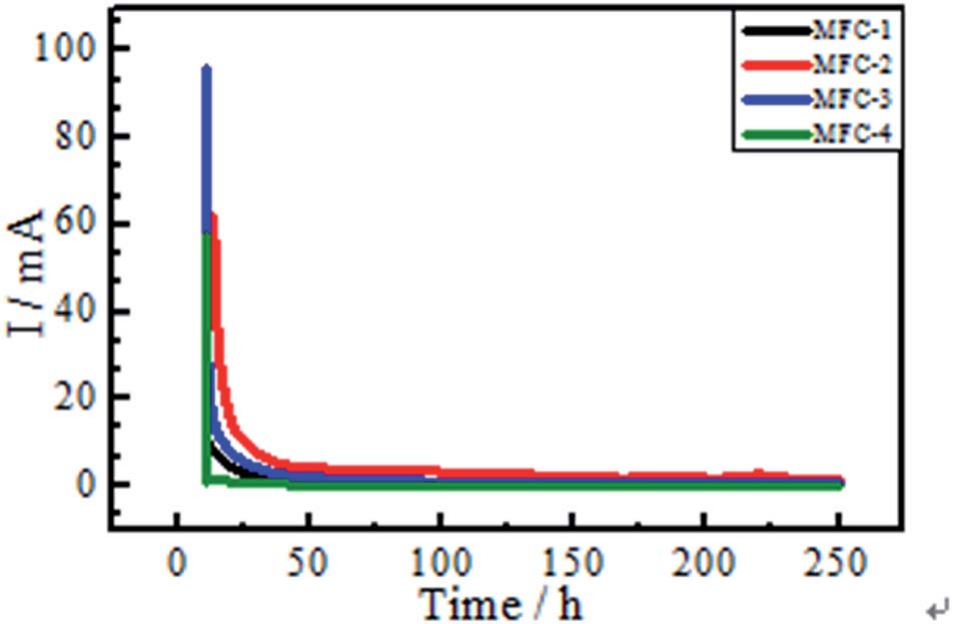
Changes of current as a function of time a constant anode potential of −0.3 V (*vs.* SCE).

Changes of current as a function of time under a constant anode potential of −0.3 V (*vs.* SCE) were shown in Fig. 4. The bioanodes exhibited electrochemical activity under a particular anode potential. The initial current values of MFC-1, MFC-2, MFC-3, and MFC-4 were fairly large, decreased sharply with time, and then reached steady currents of 0.77, 2.03, 0.69, and 0.13 mA, respectively. This result indicated that MFC-2 presented the highest bioelectrochemical activity among all MFCs.

#### Tafel analysis

**Fig. 5:**
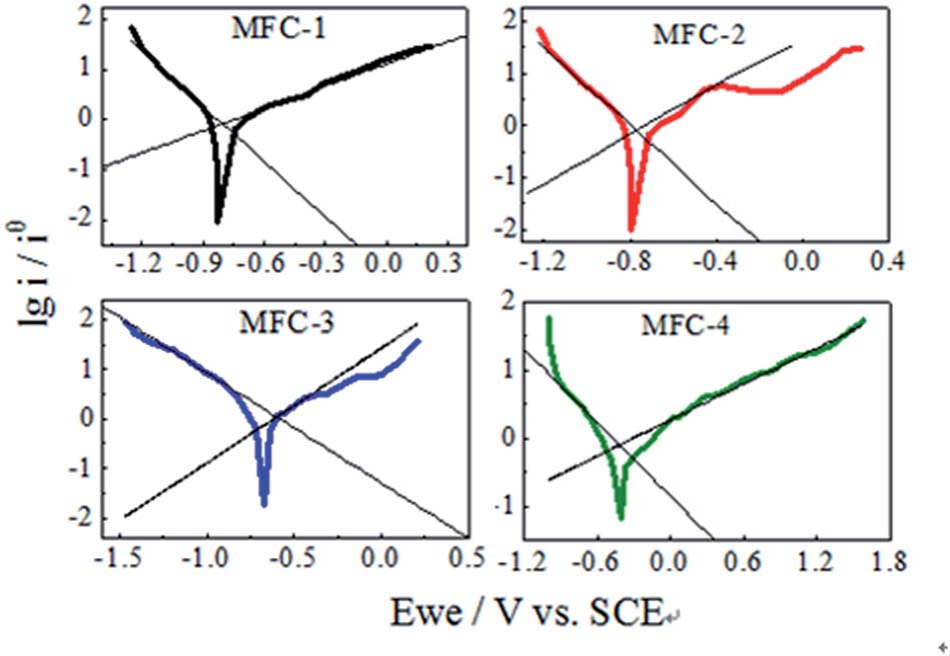
Tafel analysis of MFCs.

The bioelectrocatalytic ability of biofilm on the anodes was evaluated through Tafel slope analysis (Fig. 5). This analysis facilitated interpretation of the electrochemical activity of biofilm on carbon felt. The semi-empirical Tafel equations of oxidative and reductive reaction can be expressed as follows:

1 ln *i* = ln *i*_0_ − *α*_a_*nFE*/*RT*

2 ln *i* = ln *i*_0_ + *α*_c_*nFE*/*RT*

where *i* (mA) represents current; *i*_0_ (mA) represents exchange current; *E* (V) is applied voltage; *α*_a_, *α*_c_ represent the electron transfer coefficient of oxidative and reductive reaction, respectively; *n* is the number of electrons transmitted at the rate-limiting step; *F* is Faraday's constant (96 485 C mol^−1^); *R* is the gas constant (8.314 J mol^−1^ K^−1^); *T* is the temperature in kelvin (298 K). These equations simplify the description of kinetics of electron transfer-controlled processes to two parameters, namely, the exchange current density (*i*_0_) and Tafel slope. The Tafel slope is inversely proportional to the electrocatalytic activity and electron transfer efficiency of biocatalyst. Tafel analysis showed marked variations in electron transfer efficiency and exchange current density among bioanodes.^21^ The evaluation of biofilm activity through Tafel analysis showed a gradually decreasing oxidative slope from MFC-4 (1.134 V dec^−1^), MFC-1 (1.116 V dec^−1^), MFC-2 (0.915 V dec^−1^) to MFC-3 (0.434 V dec^−1^), meanwhile a gradually decreasing reductive slope from MFC-4 (0.559 V dec^−1^), MFC-3 (0.441 V dec^−1^), MFC-2 (0.331 V dec^−1^) to MFC-1 (0.273 V dec^−1^). Higher Tafel slope indicates the lower bio-electro catalytic activity along with electron transfer efficiencies, so MFC-4 had the worst bioelectrocatalytic activity towards oxidation and reduction comparing with the other three. According to Tafel equation, the *y*-axis intercept was logarithm of the exchange current density (ln *i*_0_). The *i*_0_ calculated at maximum performance depicted clear variations among the anodes,^22^ the values of MFC-1, MFC-2, MFC-3, and MFC-4 were determined to be 8.11, 11.97, 10.82, and 7.76 mA cm^−2^, respectively. These results clearly indicated that *i*_0_ of MFC-2 was the highest among MFCs studied.

#### Power output of MFC

**Fig. 6:**
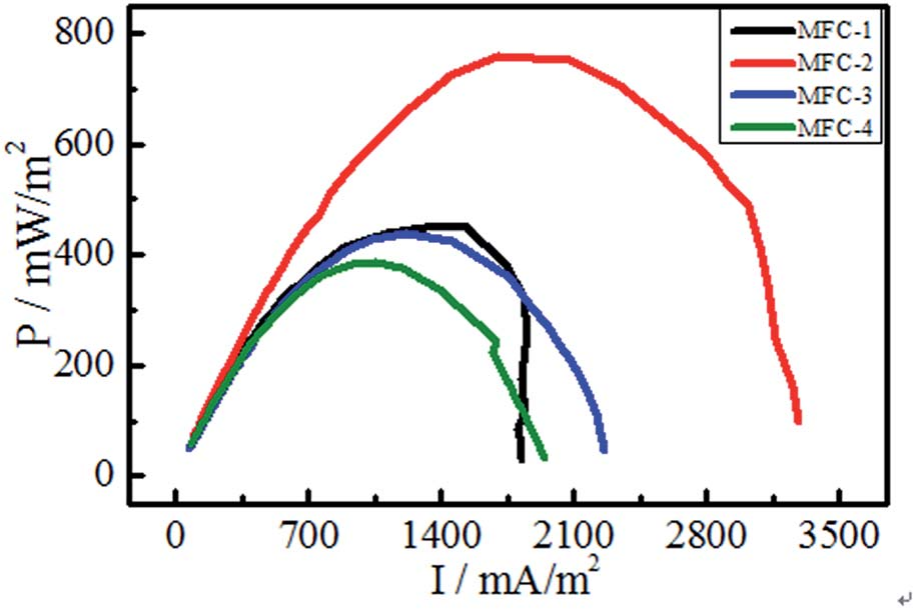
Power density curves of MFCs.

The output power densities of MFCs were shown in Fig. 6. With increasing of current density, the power density gradually increased till attaining the maximum, then decreased. Carbon felt modification significantly affected the anodic activity and power generation of MFCs. The output peak power density of MFC-1, MFC-2, MFC-3, and MFC-4 were 453.0, 758.2, 438.0, and 387.8 mW m^−2^, respectively. Among the samples, MFC-2 showed the maximum enhancement of 51.1% relative to MFC-4. No significant differences among MFC-1, MFC-3, and MFC-4 were found. The carbon felt treated with a suitable volume ratio of the mixed acids solution showed enhanced activity. Thus, we speculated that HNO_3_/H_2_O_2_ modifying could altered the physical and chemical properties of carbon felts. The modifying increased the surface roughness and specific surface area of carbon felt, which enhanced the hydrophilicity and provided a more suitable environment for bacterial growth. The carbon felt treated with volume ratio of 1 : 1 (*V*_HNO_3__ : *V*_H_2_O_2__) exhibited the best results.

#### FTIR analysis

**Fig. 7:**
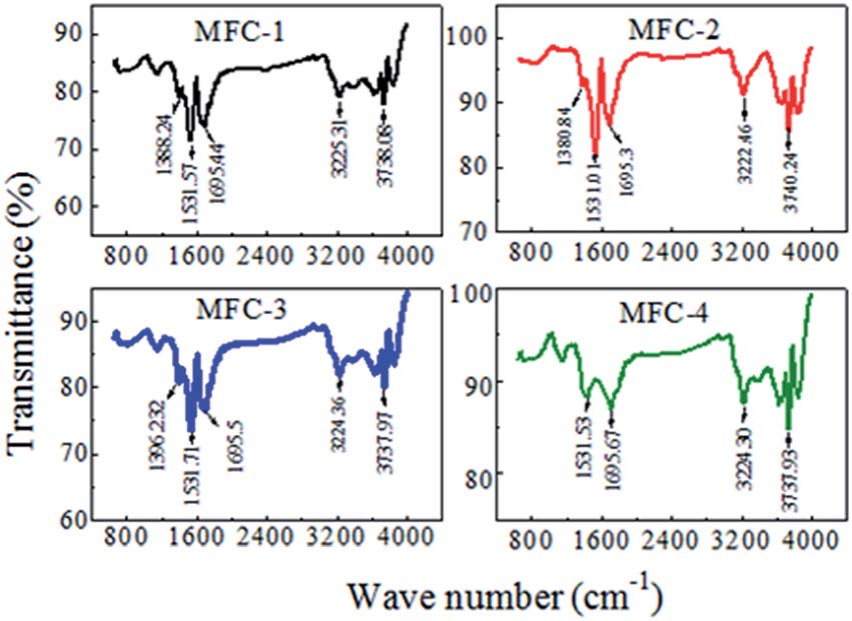
Fourier transform infrared spectra of carbon felts.

FTIR was employed to investigate the functional groups on carbon felts (Fig. 7). The untreated anode possessed a high O : C ratio, which probably resulted from the presence of surface contaminants (*e.g.*, alkaloids, resins, *etc.*) formed during fabrication. After modifying, remarkable changes in O : C ratio were observed. The relative intensity of the broad peak at approximately 3417 cm^−1^, which indicated the stretching vibrations of –OH within –COOH; and the peak at approximately 1400 cm^−1^, which indicated the bending vibrations of –OH, increased considerably. This result indicated that the quantity of –OH and –COOH increased remarkably. The peak at approximately 1710 cm^−1^, indicated the C=O stretching vibrations in –COOH, significantly increased, thus confirming that a large number of carboxyl-containing functional groups were generated. The relative intensity of the peak at 1225 cm^−1^, representing the stretching vibrations of C–O, also increased. The peak at 1597 cm^−1^ might be associated with the stretching vibrations of aromatics (C=C) and/or the bending vibration of physisorbed H_2_O.

The FTIR spectra also showed that the functional group contents varied with modifying condition. HNO_3_ was a strong oxidant that favored the introduction of carboxyl and carbonyl groups to a carbon felt, whereas H_2_O_2_ was a weak oxidant that favored the introduction of hydroxyl groups. The volume ratio of HNO_3_ : H_2_O_2_ (1 : 1) resulted in the optimal proportions of carboxyl, carbonyl groups and hydroxyl groups. Oxygen-containing functional groups on felt surfaces enhanced exoelectrogenic bacterial attachment, increased hydrophilicity, and consequently, improved electrical-chemical performance of MFCs.

### Water contact angle measurement

**Fig. 8:**
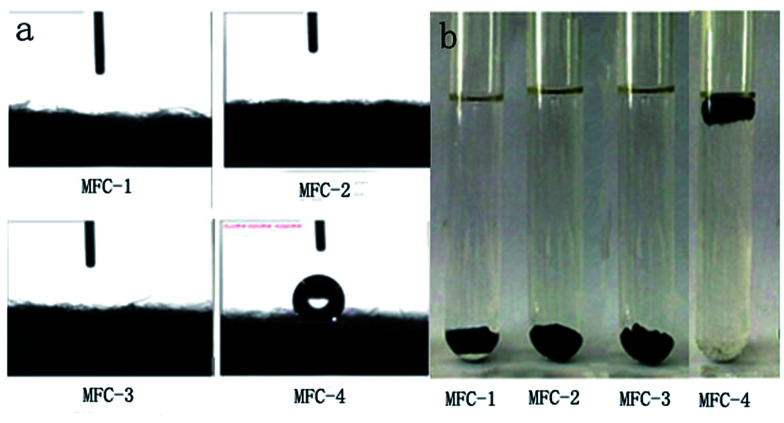
Water contact angle (a) and water absorption (b) of carbon felts.

The results of water contact angle and hydrophilicity tests of carbon felt were shown in Fig. 8(a) and (b), respectively. When the liquid (water or oil) is dropped onto a solid surface, the droplet either completely spreads or disperses at a certain angle. The included angle of the tangent from the interface of gas, liquid and solid interface is defined as contact angle. The contact angles of various solid surfaces are as follows: *θ* < 5°, super-hydrophilic/oil; 5° < *θ* < 90°, hydrophilic/oil; 90° < *θ* < 150°, hydrophobic/oil; and *θ* > 150°, super hydrophobic/oil. Most microorganisms are negatively charged by nature. Therefore, the hydrophobic/hydrophilic surfaces of the electrode affected microbial attachment and biofilm formation. From Fig. 8(a), the untreated carbon felt in MFC-4 presented a water contact angle *θ* > 120°, the distilled water drop was absorbed by the treated carbon felt fastly, however, the water contact angle was close to zero in other MFCs.^23^ The surface wettability of solid material is associated with its surface chemical composition and morphology. Therefore, we speculated that the mixed solution of HNO_3_ and H_2_O_2_ changed the surface characteristics of the carbon felts. Chemically-oxidizing remarkably reduced the water contact angles, probably owing to the increased number of the oxygen-containing functional groups on carbon felt surfaces. From Fig. 8(b), the carbon felts were placed in water for 12 h at room temperature, MFC-4 was afloat, whereas MFC-1, MFC-2, and MFC-3 sank to bottom of the vessel.^24^ This phenomenon suggested that HNO_3_/H_2_O_2_ treatment could increase the availability of hydrophilic functional groups on the surface of carbon felts. The best results was shown by MFC-2. This volume ratio of *V*_HNO_3__ : *V*_H_2_O_2__ (1 : 1) was most beneficial to hydrophilic nature of carbon felt.

### Scanning electron micrographs

**Fig. 9:**
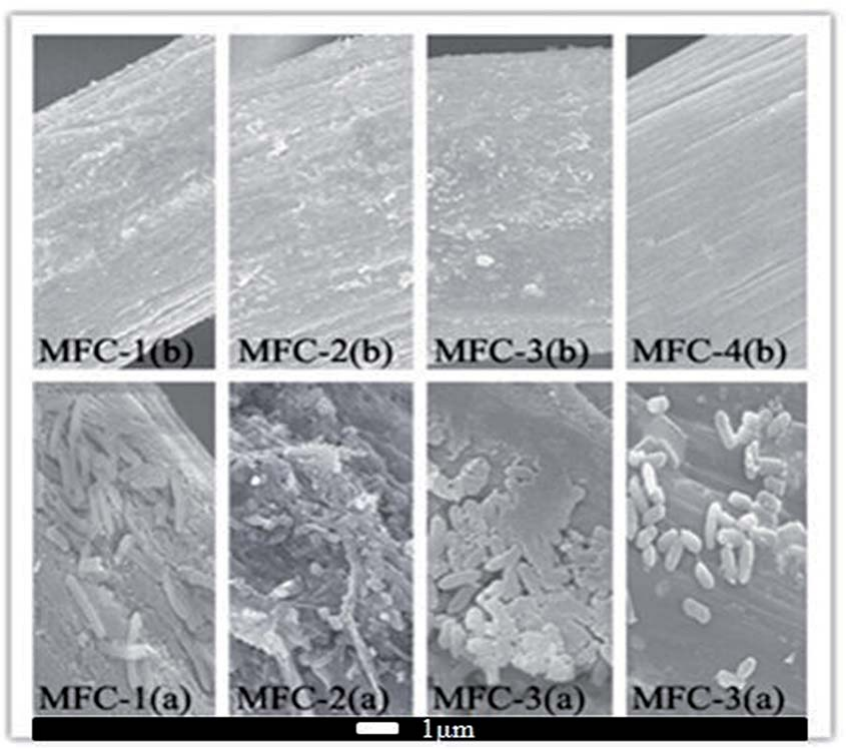
Scanning electron micrographs of carbon felts before (b) and after (a) inoculation.

Fig. 9 displayed scanning electron micrographs of carbon felts. The modified carbon felt showed varying degrees of corrosion. Modifying with increasing concentrations of HNO_3_ resulted in more rougher surface on carbon felts owing to more carbon loss, therefore provided more space for bacterial attachment. A marked change of morphology on carbon felt was caused by microbe adhering to them and colony formation, likely because oxygen-containing functional groups on the modified carbon felt surfaces significantly increased their biocompatibility. Differences kinds of functional groups presented selectivity toward different bacteria, which could lead to markedly different electrical properties of biofilms. Meanwhile more functional groups might introduce more producing-electricity bacteria adhering on carbon felt, which might promoted electron transfer from bacteria to carbon felt.^25^

## Conclusions

Carbon felts were oxidized using HNO_3_/H_2_O_2_ to increase hydrophilicity, improve biocompatibility, enhance electron transfer rates, promote the electrocatalytic properties of biofilm, especially increase the power output of the resultant MFCs substantially. Different HNO_3_/H_2_O_2_ volume ratios resulted in varying numbers and kinds of oxygen-containing functional groups. The optimal volume ratio was 1 : 1. Excessively high or low mixing proportions could result in longer start-up time, larger charge transfer resistance, lower electrochemical activity, consequently lower power output.

## Conflicts of interest

There are no conflicts to declare.

## Supplementary Material
